# Methods to Illuminate the Role of *Salmonella* Effector Proteins during Infection: A Review

**DOI:** 10.3389/fcimb.2017.00363

**Published:** 2017-08-10

**Authors:** Alexandra M. Young, Amy E. Palmer

**Affiliations:** Department of Chemistry and Biochemistry, BioFrontiers Institute, University of Colorado Boulder Boulder, CO, United States

**Keywords:** live cell imaging, fluorescence microscopy, *Salmonella* effector proteins, translocation of effector proteins, localization of effector proteins

## Abstract

Intracellular bacterial pathogens like *Salmonella enterica* use secretion systems, such as the Type III Secretion System, to deliver virulence factors into host cells in order to invade and colonize these cells. *Salmonella* virulence factors include a suite of effector proteins that remodel the host cell to facilitate bacterial internalization, replication, and evasion of host immune surveillance. A number of diverse and innovative approaches have been used to identify and characterize the role of effector proteins during infection. Recent techniques for studying infection using single cell and animal models have illuminated the contribution of individual effector proteins in infection. This review will highlight the techniques applied to study *Salmonella* effector proteins during infection. It will describe how different approaches have revealed mechanistic details for effectors in manipulating host cellular processes including: the dynamics of effector translocation into host cells, cytoskeleton reorganization, membrane trafficking, gene regulation, and autophagy.

## Introduction

**Figure 1:**
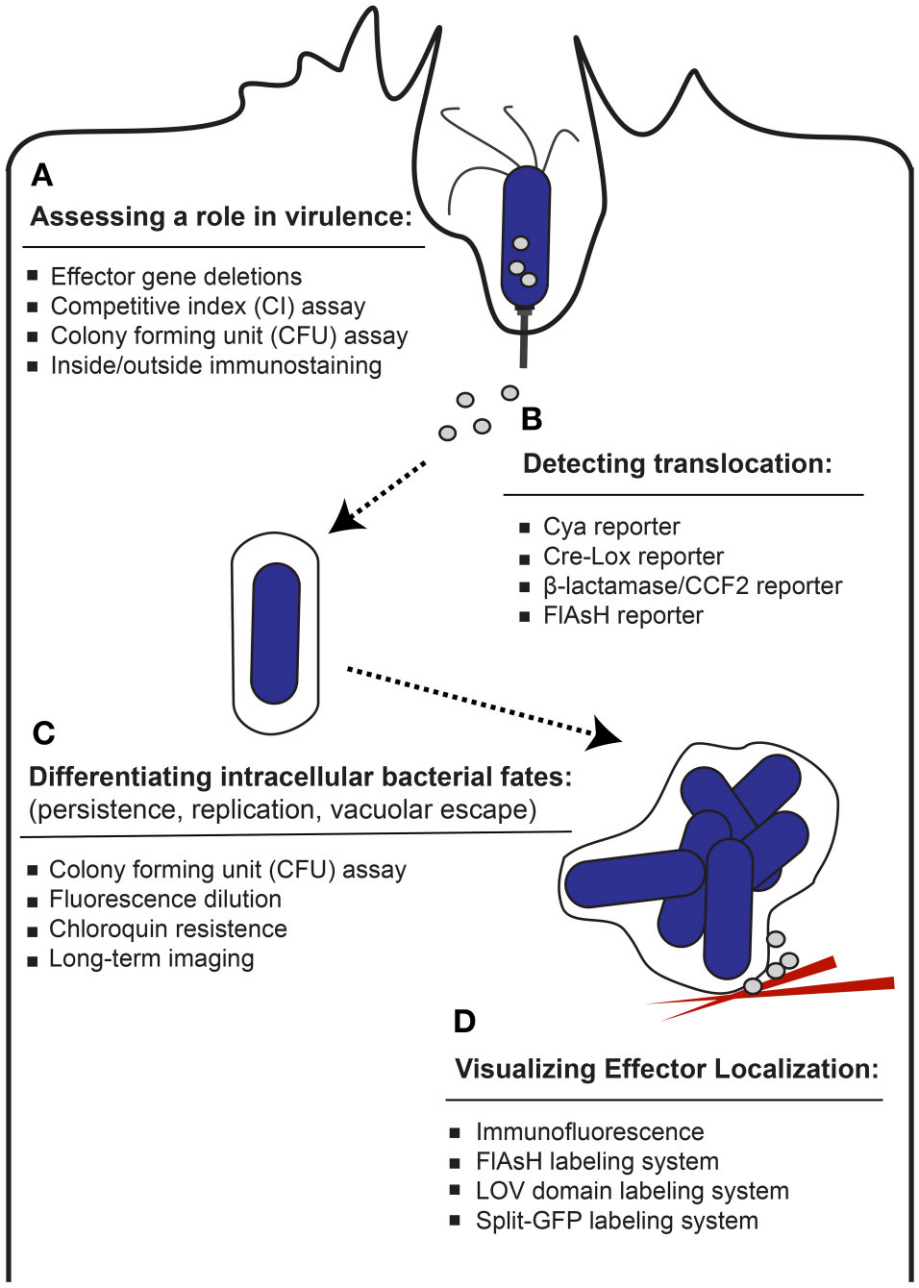
Approaches used to study effector proteins. A diverse set of techniques has been established to study different aspects of effector proteins. **(A)** Common techniques used to evaluate whether a putative effector protein plays a role in *Salmonella* infection efficiency are listed. **(B)** The methods used to report on bacterial injection of effector proteins into the host cell are listed. **(C)** The techniques used to study how an effector protein participates in the persistence, replication or vacuolar escape of internalized bacteria during the infection process are listed. **(D)** The approaches used to study effector protein localization within the host cell are listed.

Pathogenic bacteria have evolved to survive and proliferate inside of host cells despite an adverse environment driven by host defense mechanisms. Members of the Enterobacteriaceae family of pathogenic bacteria, which includes *Salmonella*, as well as *Escherichia, Yersinia, Shigella, Enterobacter*, and *Citrobacter* express specialized virulence proteins known as effectors, which are secreted into the host during the infection process. These effector proteins function to modulate the host cell by commandeering signaling pathways to enable the pathogen to invade the host, evade immune responses and establish a replication-permissive environment. One way that pathogenic bacteria, such as gram negative *Salmonella*, deliver effectors into the host cell cytosol is through specialized secretion systems such as the type III secretion system (T3SS). T3SSs evolved from the flagellar apparatus (Abby and Rocha, 2012) and represent a common mechanism for secretion of effector proteins (Marlovits and Stebbins, 2010; Moest and Méresse, 2013). *Salmonella* express two different T3SS translocons required for infection; T3SS-1, encoded by *Salmonella* Pathogenicity Island 1 (SPI-1) along with a subset of effector proteins, and T3SS-2, encoded by *Salmonella* Pathogenicity Island 2 (SPI-2) along with another cohort of effector proteins (reviewed in Malik-Kale et al., 2011). The T3SS-1 and SPI-1 expressed effector proteins generally help establish infection and play a role in bacterial uptake and generation of the *Salmonella* containing vacuole (SCV). Following internalization, a subset of bacteria is able to escape the SCV (Knodler et al., 2014) and this unique cytosolic population continues to express T3SS-1 late into infection delivering SPI-1 effectors in a second wave of translocation (Finn et al., 2017). On the other hand, the vacuolar population that persists within the SCV switch to SPI-2 expression in order to maintain intracellular life. While effector proteins are essential for enabling pathogens to establish successful infection, in many cases the functions of individual effectors and exactly how effectors promote infection are not completely understood. By determining the specific roles of these essential effector proteins in generating and sustaining an intracellular niche for bacteria, we can better understand the virulence of *Salmonella* and other Enterobacteriaceae. Therefore, innovative techniques have emerged to shed light on effector protein translocation and localization within the host cell in order to illuminate how they modulate the host cell during infection (Figure 1).

There are many methods used to identify effector proteins and to probe how they mediate the host-pathogen interface. For example, recently a sensitive method based on affinity purification (AP) followed by mass spectrometry (AP-MS) was established to identify novel host binding partners and elucidate the pathways within host cells that are targeted by effectors (Sontag et al., 2016). Many studies aim to identify the biochemical function of individual effector proteins, their role at the cellular level, and how each effector protein influences acute and chronic infection in animal models. Though biochemical-, sequence-, and structure-based studies can be instrumental in identifying and characterizing effector proteins and elucidating how they may function, these methods do not reveal information on how, when, or why an effector protein influences the infection process. Therefore, with a focus on *Salmonella*-based studies, this review will emphasize the methods developed to detect and track effector protein translocation, verify a role in virulence, and visualize localization within the host cell (Table 1).

**Table 1:** Summary of methods for studying effector proteins.

**Method**	**Resolution**	**Application**	**Purpose**	**Strengths**	**Limitations**	**References**
**TRACKING INTRACELLULAR BACTERIAL FATES**						
Competitive index (CI)	Population	Animal model and mammalian cell culture—CFU	Establishes an effector's role in virulence	Straight forward and broad applicability	Masks cell-to-cell heterogeneity, No mechanistic detail	Beuzón et al., 2002; Knodler et al., 2003; Deiwick et al., 2006; Henry et al., 2006; Sieuwerts et al., 2008; Figueiredo et al., 2009; Santiviago et al., 2009
Chloroquine resistance	Population	Mammalian cell culture—CFU	Establishes an effector's role in vacuolar escape	High selectivity	Limited to infection models with cytosolic bacteria	Knodler et al., 2014
Immunofluorescence (IF) (inside/outside staining)	Single cell	Mammalian cell culture—Fixed cell microscopy	Distinguishes adhered from intracellular bacteria to establish an effector's role in bacterial uptake	High sensitivity	Limited to early stages of infection	Zhou et al., 1999; Dai et al., 2004; Lara-Tejero and Galán, 2009; Misselwitz et al., 2012; Li et al., 2013
Replication reporter based on fluorescence dilution	Single cell	Mammalian cell culture—Live cell microscopy, FACS	Monitors replication efficiency to establish an effector's role in bacterial persistence and growth	Quantitative, Direct measure of replication	Tracks replication up to 10 generations	Helaine et al., 2010
Long-term imaging	Single cell	Mammalian cell culture—Live cell microscopy	Tracks an effector protein's contribution to replication, persistence and vacuolar escape	Captures multiple phenotypes in 1 experiment	Relatively low throughput, Requires a microscope equipped with an environmental chamber and autofocus	McQuate et al., 2017
**DETECTING EFFECTOR PROTEIN TRANSLOCATION**						
Cya reporter	Population	Bacterial culture and mammalian cell culture–Immunoassay	Detects the presence of an effector translocated into media or the host cell by cAMP production	Broad applicability	Indirect read-out, Requires previous knowledge of translocation time scale	Sory and Cornelis, 1994; Briones et al., 2006
Cre-Lox reporter	Population or Single cell	Mammalian cell culture—Microplate reader, live cell microscopy and FACS	Detects the presence of an effector translocated into the host cell by initiation of GFP expression (or firefly luciferase)	Well suited for screening	Not quantitative	Briones et al., 2006
β-lactamase/CCF2 reporter	Population or Single cell	Animal model and mammalian cell culture—Live cell microscopy and FACS	Detects the presence of an effector translocated into the host cell through color change due to cleavage of a FRET pair	High sensitivity	Not quantitative, No real time studies	Charpentier and Oswald, 2004; Geddes et al., 2007; Schlumberger et al., 2007; Sun et al., 2007; Newton et al., 2016
**VISUALIZING EFFECTOR PROTEIN TRANSLOCATION AND LOCALIZATION IN THE HOST CELL**						
FlAsH labeling system	Single cell	Mammalian cell culture—Fixed or Live cell microscopy	Visualization of an effector's translocation through the depletion of bacterial fluorescence or Visualization of an effector's localization at fixed time points or over time	Direct effector labeling, Small label size, Real time kinetics	Limited to early translocated effectors for visualizing translocation (T3SS-1 effectors). Low sensitivity due to limited signal intensity and background fluorescence, Toxicity has to be tightly controlled, Potential perturbation of effector function due to tag	Griffin et al., 1998; Enninga et al., 2005; Van Engelenburg and Palmer, 2008
Immunofluorescence (IF)	Single cell	Mammalian cell culture—Fixed cell microscopy	Visualization of an effector protein's localization at fixed time points	No modification of host	Unable to capture dynamic processes, Fixation can alter host membrane structures, Few antibodies available against effectors requiring most to be epitope tagged	Beuzón et al., 2000; Zhou et al., 2001; Brumell et al., 2002; Hernandez, 2004; Kuhle et al., 2004; Birmingham et al., 2005; Bujny et al., 2008; Mallo et al., 2008; Ohlson et al., 2008; Knodler et al., 2009, 2010; Patel et al., 2009; Schroeder et al., 2011; Choi et al., 2013
LOV domain labeling system	Single cell	Mammalian cell culture—Live cell microscopy	Visualization of an effector's translocation and localization over time	Direct effector labeling, Real time kinetics, uses cellular flavins so no need for additional dyes	Low sensitivity due to limited signal intensity, Potential perturbation of effector function due to tag	Gawthorne et al., 2012, 2016
Split-GFP labeling system	Single cell	Mammalian cell culture—Live cell microscopy	Visualization of an effector's localization at fixed time points or over time	Direct effector labeling, Long-term visualization in real time	Complementation kinetics limit use to 2 h P.I. and after, Moderate sensitivity due to limited signal intensity, Potential perturbation of effector function due to tag	Cabantous et al., 2005; Cabantous and Waldo, 2006; Van Engelenburg and Palmer, 2010

## Detection of effector protein translocation into host cells

**Figure 2:**
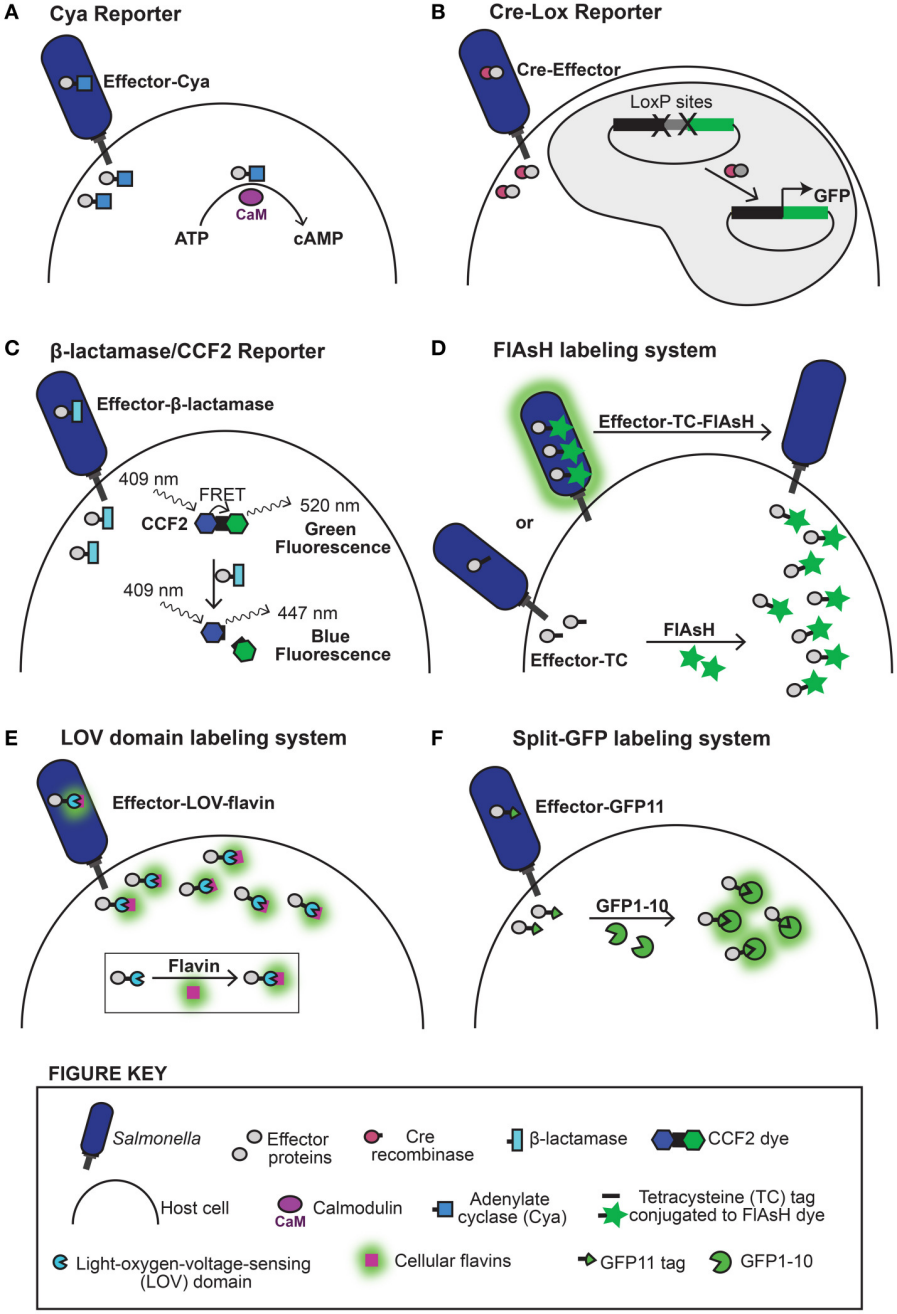
Methods for detecting effector protein translocation and host cell localization. Approaches used to detect effector translocation are schematized **(A–D)**. **(A)** The Cya reporter system uses the enzyme calmodulin-dependent adenylate cyclase (Cya) (represented by a blue box) fused to an effector protein to detect translocation. Injection of the effector-Cya fusion protein into host cells during infection generates detectible increases in cyclic AMP (cAMP) following reaction with host cell calmodulin (represented by a fuchsia oval). **(B)** The Cre-Lox reporter detects effector translocation through recombination driven expression of GFP (or firefly luciferase). This system consists of a bacterial effector protein fused to Cre recombinase (represented by a purple circle) and host cells expressing a LoxP reporter plasmid. Following effector translocation, the LoxP sites (represented by X's) are cleaved and transcription of the GFP reporter is triggered resulting in fluorescent host cells. **(C)** The β-lactamase/CCF2 reporter system uses enzymatic detection where fluorescence excitation of the CCF2 dye coumarin moiety (represented by a blue hexagon) at 409 nm results in fluorescence resonance energy transfer (FRET) to the fluorescein moiety (represented by a green hexagon), which emits a green fluorescence signal at 520 nm. Translocation of an effector protein fused to β-lactamase (represented by a turquoise rectangle) into a CCF2-loaded host cell induces cleavage of the CCF2 β-lactam ring (represented by a black square), abolishing FRET and producing a detectable change in fluorescence emission from green to blue (447 nm). **(D)** The FlAsH labeling system uses a tetracysteine (TC) motif (represented by a black bar) that selectively binds to the biarsenical dye FlAsH (represented by a green star) to produce a fluorescent label. The FlAsH labeling system is used to track effector protein translocation by monitoring depletion of bacterial fluorescence in real time. Bacteria expressing an effector protein tagged with a tetracysteine (TC) affinity motif are treated with the biarsenical dye, FlAsH (represented by a green star), which selectively binds the TC tag prior to infection generating fluorescently labeled bacteria. Translocation of the fluorescently labeled effector protein upon infection results in detectable depletion of the bacterial fluorescence signal as the labeled effector protein is injected into the host cell where the signal becomes too diffuse to detect further. Approaches used to study the host localization of translocated effector proteins are schematized **(D–F)**. **(D)** When effector proteins tagged with the TC motif are translocated into a host cell loaded with FlAsH dye the two components combine to produce a fluorescently labeled effector protein that can then be visualized. **(E)** The Light-oxygen-voltage-sensing (LOV) domain (represented by a blue semicircle) is a light-sensing motif that binds the chromophore flavin mononucleotide (represented by a purple box) to emit green fluorescence when irradiated with blue/UV light. Effector proteins fused to the LOV domain will bind cellular flavins within bacteria to generate a fluorescent label that can be tracked during and after translocation into the host cell. **(F)** The split-GFP labeling system uses fluorescence complementation that occurs between effector proteins tagged with GFP11 (represented by a green wedge) and GFP1-10 (represented by a green semicircle) expressed in the host cell. Infection and translocation of GFP11-labeled effector proteins leads to spontaneous joining of the two components to generate a full GFP fluorescent label (represented by a green circle).

A wide variety of methods ranging from bioinformatics and biochemistry to live cell imaging have been used to identify effector proteins and probe how they mediate infection (reviewed in Ramos-Morales, 2012). To detect whether a putative effector protein is translocated into a host cell several reporter systems have been developed. These reporter constructs were designed to overcome challenges posed by direct detection of lowly abundant effector proteins that are only secreted upon infection of host cells. Candidate effectors can be expressed under constitutive promoters with reporters that improve detection for facile screening. The calmodulin-dependent adenylate cyclase domain, derived from the cyclolysin (CyaA) toxin from *Bordetella pertussis*, is routinely used as a translocation reporter (Sory and Cornelis, 1994). This reporter approach works by fusing the N-terminal portion of an effector protein (which is often sufficient to direct substrate translocation through the T3SSs), or in some cases the full effector protein, to the catalytic adenylate cyclase domain of CyaA (Figure 2A). If bacteria translocate the resulting effector-CyaA hybrid protein into the cytosol of host cells, it will bind to calmodulin and produce a detectable accumulation of cyclic AMP (cAMP) from ATP. Because calmodulin is not commonly encoded by bacteria, CyaA is not active prior to translocation and it is not naturally translocated by the T3SSs. Therefore, an increase in cytosolic cAMP levels in a host cell is indicative of T3SS-dependent translocation of the CyaA reporter because calmodulin is only present in the cytosol of eukaryotic cells. The CyaA system has been widely exploited (Sory and Cornelis, 1994; Miao et al., 1999; Miao and Miller, 2000; Kujat Choy et al., 2004; Geddes et al., 2005; Niemann et al., 2010) to report on the translocation of effector proteins. Typically cell lysates are assessed using enzyme-linked immunosorbent assays (ELISA) that offer high sensitivity, low limits of detection, and robust separation between positive and negative substrate translocation. However, to capture transient and reversible changes in the level of cyclic AMP within the host cell may require sampling of time frames up to 21 h post infection (Kujat Choy et al., 2004). This is because a subset of effector proteins is translocated immediately upon contact with host cells, where others are translocated hours later following bacterial internalization, and thus the timing of individual effector protein secretion may vary with different stages of infection.

A complementary method for detecting translocated bacterial effectors using microscopy or fluorometry is based on the use of the bacteriophage P1 Cre-Lox system to generate a fluorescence or luminescence signal upon delivery of the effector into the host cell (Briones et al., 2006; Figure 2B). The Cre enzyme catalyzes recombination between *loxP* sequences. This Cre system was used to demonstrate the T3SS-1 dependent translocation of *Salmonella* effector protein SopE upon contact with host cells (Briones et al., 2006). The first 104 amino acids of SopE were fused to the full length Cre recombinase and translocation was assessed by Cre mediated excision of intervening sequences on a firefly luciferase or green fluorescent protein (GFP) reporter expressed within the host cell.

Another approach for detecting effector protein translocation using microscopy or fluorometry uses a β-lactamase/CCF2 based reporter system (Zlokarnik et al., 1998; Charpentier and Oswald, 2004) (Figure 2C) that enables a direct readout (Mills et al., 2008). This approach involves fusion of β-lactamase to an effector protein of interest and the introduction of a freely diffusing dye (CCF2) that undergoes a color-change upon hydrolytic cleavage by β-lactamase into the host cell. Pretreatment of mammalian cells with CCF2 prior to infection enables the system to report on the delivery of effector proteins into the host cytosol upon infection due to the different color of the cleavage product which can be detected in live cells using fluorometry or fluorescence microscopy. Though there may be inherent background signal due to some CCF2 cleavage in uninfected cells, this approach has been used to demonstrate the different cell types targeted by *Salmonella* in a mouse model of infection (Geddes et al., 2007), to indicate the translocation of bacterial flagellins which are potent inducers of innate immunity (Sun et al., 2007), as well as to ensure translocation of genetic variants of the effector protein SipA while probing for functional domains using deletion analysis (Schlumberger et al., 2007).

An approach for visualizing effector proteins within bacteria prior to translocation is the FlAsH/tetracysteine labeling system (Enninga et al., 2005; Van Engelenburg and Palmer, 2008) (Figure 2D). This system uses the fluorescein-based biarsenical dye (FlAsH), which binds a 15 amino acid tetracysteine (TC) motif that can be appended to an effector protein for detection. The unbound FlAsH dye is weakly fluorescent and undergoes a large increase in fluorescence signal upon coordination to the tetracysteine motif (Griffin et al., 1998). The FlAsH labeling system was used to visualize real time effector protein translocation into host cells upon infection by monitoring the depletion of effectors from the bacterial cytosol (Enninga et al., 2005; Van Engelenburg and Palmer, 2008). This technique was used to demonstrate that two *Salmonella* effector proteins, SopE2 and SptP, exhibit different secretion kinetics, revealing a kinetic hierarchy for effector translocation into host cells (Van Engelenburg and Palmer, 2008). Additionally, because this system involves a physically tethered fluorescent label, it can be used to monitor effector proteins before and throughout the translocation process. However, poor signal to noise limits the use of this system in visualizing diffuse effector protein populations in live host cells.

It should be noted that imaging approaches used for effector protein detection may require sophisticated equipment outfitted with appropriate filter sets and necessitate capturing many (>100) cells sampled at random in order to achieve statistical significance.

## Methods to visualize effector proteins in fixed host cells

**Figure 3:**
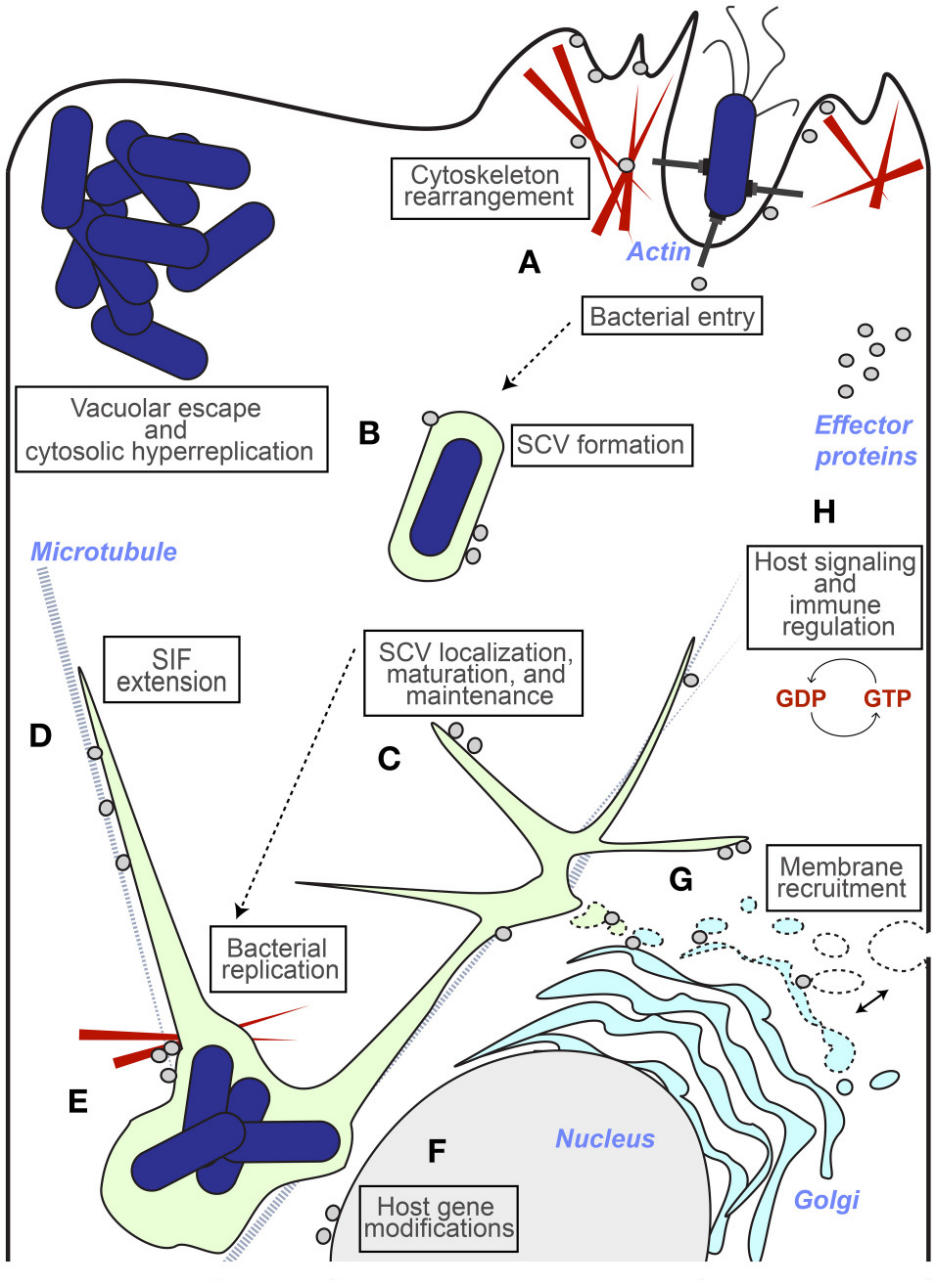
Insights from effector protein localization. The different host cell destinations for effector proteins (represented by gray circles) that have provided insight into function during infection are represented. **(A)** Effectors that have been shown to localize to the ruffling host cell membrane (or to host cell factors localized here) and polymerizing actin near invasion sites have roles in cytoskeleton rearrangements that lead to bacterial entry (e.g., SipA, SopB, SopE, SopE2, SptP). **(B)** Effectors that localize to the SCV have roles involving SCV formation and maintenance, (e.g., SifA, SopB, SseF, SseG, SteA) and their presence or absence contributes to vacuolar escape by bacteria and subsequent cytosolic hyperreplication (e.g., SseG and SifA respectively). **(C)** Effector localization at the SCV also includes roles in SCV localization and maintenance, which contributes to bacterial replication efficiency (e.g., SteA, SseG). **(D)** Effectors that contribute to vacuole membrane dynamics through the regulation of *Salmonella* Induced Filaments (SIFs) localize to these extensions and have been shown to associate with microtubules and motor proteins (e.g., SifA, PipB2, SopD2). **(E)** Effectors that localize to the SCV and F-actin meshwork (represented by red wedges) that forms around it have a role in anchoring the SCV in a perinuclear position (e.g., SseI, SspH2, SteC). **(F)** Modulation of host gene expression is accompanied by a nuclear localization (e.g., SpvC, SspH1). **(G)** The recruitment of host membrane to the maturing SCV is accomplished by effectors that associate with SIFS and host endocytic and exocytic traffic (e.g., PipB2, SseF, SseG, SteA). **(H)** Effectors that interfere with host signaling pathways and immune regulation have access to host signaling factors in the cytosol (e.g., GogB, GtgE, SopD, SpvC, SseL).

The localization of effector proteins within the host cell at different stages of infection is important considering how the pathogen manipulates host cell processes in different subcellular regions (Figure 3). Defining where and when an effector protein is localized, and how localization may change over time, can highlight that protein's role in the infection process at the cellular level. Approaches involving fixing and staining infected host cells or tissue slices at discrete time points post infection can be used to address the localization of effector proteins within the context of infection. Because these assays allow for the visualization of *Salmonella* within the host cell in relation to effector proteins or host cell markers, they have the potential to provide information about effector protein functions during infection. For example, the effector protein SopB has been shown to play unique roles at different stages of infection that correspond to different subcellular localizations. At initial stages of infection, SopB resides at the host cell membrane ruffling events and functions to promote membrane fusion following bacterial internalization (Zhou et al., 2001; Hernandez, 2004). At later stages, however, SopB relocates from the plasma membrane to the SCV by an ubiquitin dependent mechanism (Knodler et al., 2009; Patel et al., 2009). This change in localization helps explain another role for SopB in promoting SCV maturation and tubule formation through the recruitment of Rab5 and modulation of lipid content on the SCV (Mallo et al., 2008). Thus, defining the localization of effector proteins within the host cell at different stages of infection provides insight into how the pathogen manipulates host cell processes in different subcellular regions.

Immunofluorescence-based visualization of effector proteins relies on antibodies conjugated to fluorophores which recognize either an epitope-tagged effector protein or an effector itself. Because there are very few antibodies against individual effector proteins, immunofluorescence studies typically involve detection of an epitope-tagged version of the effector of interest, such as the HA-tag or FLAG-tag. Immunofluorescence approaches have shown that SCV-associated filaments are diverse, based on the accumulation of different effector proteins and recruitment of different host cell markers (Schroeder et al., 2011). Immunofluorescence has also been essential for defining the involvement of specific effector proteins in established *Salmonella* infection phenotypes such as the roles of SopB in the recruitment of sorting nexin-1 to the SCV (Bujny et al., 2008), SifA, SseJ, SseG, and SseF in tubule formation (Beuzón et al., 2000; Kuhle et al., 2004; Birmingham et al., 2005), the role of SptP in allowing *Salmonella* to spread between organs within the mouse (Choi et al., 2013), the role of SPI-1 (but not SPI-2) in promoting escape from the SCV (Knodler et al., 2010), and the roles of SifA, SseJ, and SopD2 in SCV membrane integrity (Brumell et al., 2002; Ohlson et al., 2008).

The FlAsH/tetracysteine labeling system introduced above for visualizing translocation of effector proteins can also be used for monitoring effector proteins within host cells (Griffin et al., 1998; Enninga et al., 2005; Van Engelenburg and Palmer, 2008; Figure 2D). By loading the host cell with FlAsH following infection, this labeling system was used to show that the *Shigella flexneri* T3SS effector proteins IpaB and IpaC localize to actin foci at invasion sites in fixed cells (Enninga et al., 2005), however the signal to noise wasn't high enough to track Salmonella effectors in live cells (Van Engelenburg and Palmer, 2008).

The approaches described above for visualizing effector proteins in fixed cells are powerful tools for revealing spatial relationships between *Salmonella*, effector proteins and the host environment. However, images at fixed time points can't capture dynamics, and make it challenging to capture phenotypes that evolve over time, such as the recently described dispersion of the SCV at early time points and coalescence at later time points (McQuate et al., 2017). Furthermore, cell fixation has the potential to alter infection phenotypes, such as the integrity of the membrane that composes SCV filaments (Rajashekar et al., 2014).

## Live cell methods to visualize effector proteins in host cells

There is a growing need to develop new tools that capture and highlight effector protein localization in live infected cells in order to unravel specific effector protein roles in a spatial and temporal context of infection while preserving cell-to-cell heterogeneity that is apparent in single cell studies. Live cell imaging approaches allow for the observation of cellular events unfolding in real time, and are therefore desirable for elucidating dynamic processes. However, monitoring bacterial effector proteins during the infection of live cells is technically challenging due to the mechanism of effector protein translocation through the T3SS into the host cell. Effector proteins are escorted and unfolded by chaperones in the bacterial cytosol in order to be threaded through the needle-like T3SS translocon for transport into the host cell, where the effectors are then refolded following delivery into the host cytosol (Akeda and Galán, 2005; Tsai et al., 2015). This process of threading through the translocon is incompatible with fluorescent protein (FP) tagging due to the high thermodynamic stability of FPs (Radics et al., 2014). Therefore, tagging and visualizing T3SS translocated bacterial effector proteins during live cell infections relies on alternate labeling approaches. Several established techniques make use of small affinity tags that label an effector protein within bacteria coupled with complementary components that are either introduced to the bacteria or to a host cell to generate a fluorescent label when the two components join together.

One system that is capable of monitoring the fate of translocated effector proteins within living host cells during infection uses a light-oxygen-voltage-sensing (LOV) domain (Figure 2E). When conjugated to an effector protein of interest the LOV-domain functions as a reporter that binds to cellular flavin mononucleotides to produce a fluorescent tag. This LOV-domain technology has been used to monitor real time effector protein expression and translocation, as well as to track effector localization upon introduction into the host cell (Gawthorne et al., 2012, 2016). The-LOV domain reporter system remains ideal for capturing early events in infection and was used to track the *Shigella flexneri* effector protein IpaB, which was shown to localize preferentially at bacterial poles before rapid translocation and final localization at the bacterial entry site within membrane ruffles (Gawthorne et al., 2016). However, with a relatively low quantum yield (0.2–0.4; Buckley et al., 2015), the LOV-domain reporter may not be ideal for visualizing all effector proteins because some *Salmonella* effectors have been shown to express and translocate at low levels (Xu and Hensel, 2009).

The only other live cell approach currently available for visualizing translocated effector protein localization within the host cell is based on fluorescence complementation using the split-GFP system (Van Engelenburg and Palmer, 2010; Young et al., 2017; Figure 2F). Split-GFP is composed of two fragments of the GFP β-barrel that were engineered to be stable, soluble, and non-fluorescent in isolation and to combine spontaneously and irreversibly to form the GFP chromophore and recapitulate GFP fluorescence (GFP_comp_) (Cabantous and Waldo, 2006). To exploit the split GFP system for effector protein tagging, the small 13-amino-acid 11th strand of the GFP β-barrel (GFP11) is genetically fused to *Salmonella* effector proteins. The complementary strands of GFP (GFP1–10) are expressed *in trans* in the host cell prior to infection and upon challenge with *Salmonella* and T3SS effector translocation, spontaneous complementation of the two split-GFP fragments results in fluorescent tagging and visualization of the effector population within the host cell. The split-GFP labeling system is best suited for visualization of effector proteins at later time points post infection (from 2 to 24+ h) due to the time required for fluorescence complementation (Cabantous et al., 2005). The split-GFP system was therefore adapted for labeling T3SS-2 effectors. This approach enabled the visualization of *Salmonella* effector proteins SteA, SteC, and PipB2 in epithelial cells, and PipB2 in the macrophage cell line RAW264.7, illustrating the usefulness of split-GFP in tagging diverse T3SS effectors and tracking effector populations in live host cells over time (Van Engelenburg and Palmer, 2010). Recently, split-GFP was expanded for use in primary bone marrow derived macrophage cells and revealed distinctly different localization phenotypes for PipB2 and SteA in epithelial cells compared to immunocompetent primary macrophages (Young et al., 2017). This study suggests that different types of host cells provide unique environments for *Salmonella*, which potentially corresponds to different roles for effector proteins and underscores the importance of studying multiple infection models.

## Methods to assess the role of effector proteins in virulence

The ability of *Salmonella* to influence the fate of the host cell is an important part of the infection process. In addition to forming and maintaining the intracellular niche, effector proteins are able to regulate host cell immune signaling processes and host cell viability in order to benefit the intracellular fate of *Salmonella* (reviewed in Ramos-Morales, 2012). One commonly used technique to examine effector virulence functions, is to infect cells or model organisms with strains of *Salmonella* lacking the effector protein. Such studies seek to define and determine changes to infection phenotypes compared to the wild type strain in order to gain insight into an effector protein's function during infection. These differences may include the level of *Salmonella* invasiveness, the ability of bacteria to replicate, persist and disseminate within an organism or cell, or more specific features of infection at the cellular level such as perturbation of cellular organelles, location of the SCV or bacteria within the host cell and host inflammatory responses.

Infection models are key for studying the role of effector proteins, however, it is important to recognize the nuances associated with different model systems in order to understand the effects of specific effectors in the model compared to natural hosts (Haraga et al., 2008). Mice have served as the dominant model system for studying *Salmonella* infection at the animal level but *Salmonella* infection in mice does not always mimic the diseases presented in humans. For example, *Salmonella* Typhimurium, which infects a broad range of animal hosts, causes inflammatory enteritis in humans but results in systemic Typhoid-like disease in mice (Gal-Mor et al., 2014). In humans, Typhoid disease is caused by *Salmonella* Typhi, which is restricted to human hosts. Therefore, different approaches have been developed that modify the mouse model to better reflect human infection. Mice pre-treated with Streptomycin, for example, will manifest acute intestinal infection including inflammation and diarrhea when infected with *Salmonella* Typhimurium (Barthel et al., 2003). This model enables the use of mice to study acute intestinal disease and the role of specific T3SS effector proteins in enteritis. The most extensively used mouse model for investigating the contribution of effector proteins in infection is the natural-resistance-associated macrophage protein 1 (Nramp1)-null mouse model (Hormaeche, 1979). These mice are immunocompromised, susceptible to mortal infection, and have been successfully used to identify many T3SS-associated genes important for infection (Hensel et al., 1998; Beuzon and Holden, 2001). Nramp1 is a macrophage specific ion transporter that exports ions from the SCV, starving bacteria of nutrients and limiting bacterial replication. Nramp1-null mice do not survive long enough to study effectors important in maintaining persistent infection. The Nramp1-positive mouse model is therefore necessary to study long-term systemic infection and investigate how different effector proteins contribute to persistence (Monack et al., 2004; Lawley et al., 2006). Studies with Nramp1-positive mice have revealed a role for the SPI-1 expressed T3SS-1 in systemic disease (Galán and Curtiss, 1989) and confirmed that SPI-2 effectors are important for bacterial colonization and maintaining persistent infection (Behlau and Miller, 1993). The proper selection of an animal model system can be critical for defining the physiologically relevant roles of effector proteins as the diversity and evolution of the effector content of *Salmonella* strains can vary, in part due to selection for the presence or loss of individual effectors in specific animal populations or disease settings (Haraga et al., 2008).

One of the hallmarks of successful *Salmonella* infection in Nramp1-positive mouse models is a persistent infection that breaches the small intestine and spreads to other organs. A primary method used to examine the role of an effector protein in virulence during this mouse model of infection is called a competitive index (CI) assay. In a CI assay, strains of WT *Salmonella* are pitted against strains lacking the effector protein of interest, and both strains are used simultaneously to infect a live mouse (Hensel et al., 1995; Lawley et al., 2006; Santiviago et al., 2009). Comparing how both strains fare in a single mouse controls for mouse-to-mouse variability. Infected mice are sacrificed at 2–4 days post infection and organs are examined for the presence of *Salmonella* by colony forming units (CFUs). For CFU assessment, the organ lysate is plated on agar with appropriate antibiotics for each strain and incubated for bacterial growth. The number of colonies recovered is proportional to the bacterial load at a particular time point and is indicative of each strain's invasion or replication ability (Sieuwerts et al., 2008). The CFU results are used to indicate which strain fared better within the mouse and reveal whether or not the effector protein had an impact on fitness (Santiviago et al., 2009). The CI/CFU assay is a useful starting point for investigating the role of an effector protein during infection. By incorporating a time parameter, the CFU approach can also be used to differentiate between a role in invasion or replication, as both of these processes increase the bacterial load within cells. Cells or tissues assessed at 1–2 h post infection reveal invasion efficiency (Henry et al., 2006; Figueiredo et al., 2009), whereas 6–22 h post infection are used to indicate replication efficiency (Beuzón et al., 2002; Knodler et al., 2003; Deiwick et al., 2006).

Although, the CFU assay is useful in establishing whether an effector protein plays a general role in promoting *Salmonella* virulence, it fails to show invasion or replication on the single cell level and can therefore mask cell-to-cell heterogeneity. For example, *Salmonella* can display different infection phenotypes from one cell to the next due to the use of different invasion mechanisms for individual epithelial cells (Rosselin et al., 2010; Velge et al., 2012; Rajashekar et al., 2014), the ability to replicate inside the SCV or escape and hyper-replicate in the cytosol of epithelial cells (Knodler et al., 2010), and the propensity to experience different fates in macrophage cells (Helaine et al., 2010; McQuate et al., 2017). These cell-to-cell variations in infection phenotypes may represent the differential presence and function of effector proteins (LaRock et al., 2015). To accommodate this heterogeneity, complementary methods have been developed to examine invasion and replication phenotypes on the single cell level. For example, a differential “inside/outside” immunostaining method (Chen et al., 1996; Zhou et al., 1999, 2001; Dai et al., 2004; Lara-Tejero and Galán, 2009; Misselwitz et al., 2012) can be used to determine invasion efficiency. For the inside/outside assay, cells are fixed with paraformaldehyde at discrete time points post infection (often 15 min to 1 h). The extracellular bacteria are stained using fluorescently labeled antibodies prior to host cell membrane permeabilization. Following membrane permeabilization, host cell markers and internalized bacteria may be labeled with differently colored probes so that all bacteria are singly labeled and only extracellular bacteria are doubly labeled. Thus, upon visualization of infected cells using fluorescence microscopy, extracellular bacteria may be enumerated as they are clearly differentiated from intracellular bacteria and the internalization efficiency of mutant strains can be scored (Zhou et al., 1999; Dai et al., 2004; Li et al., 2013). This method was used by Zhou et al. to define a critical role for the actin-binding effector protein SipA in bacterial internalization (Zhou et al., 1999), and by Lara-Tejero and Galán to demonstrate that bacterial adherence to nonphagocytic host cells requires the T3SS-1 translocon (Lara-Tejero and Galán, 2009).

## Methods to monitor intracellular bacterial fates: persistence, replication, and vacuolar escape

Intracellular bacteria can proliferate, persist or be subjected to killing over the course of infection and these processes are difficult to distinguish. The fate of bacteria is often assessed through CI/CFU assays that determine net bacterial load, which is the product of both replication and death undergone by the population. However, this measurement of net bacterial load can't distinguish defects in replication from increased incidence of bacterial killing, and masks heterogeneity within bacterial populations. This distinction has been shown to be particularly important in persistent infections in which slow or non-growing bacteria are thought to have a major impact (Helaine et al., 2010).

To directly measure bacterial replication and enable visualization of the heterogeneity of intracellular bacterial populations Helaine et al. (2010) developed a reporter system based on fluorescence dilution that permits direct quantification of the replication dynamics of *Salmonella* at both the population and single-cell level. This dual fluorescence reporter functions by measuring a pre-formed pool of arabinose induced DsRed protein in replicating bacteria also expressing EGFP constitutively or by isopropyl β-D-thiogalactoside (IPTG) induction. Upon each bacterial division event in the absence of arabinose, DsRed fluorescence signal intensity is halved. Therefore, as the bacterial population replicates DsRed fluorescence undergoes a signal dilution that can be monitored and the magnitude of the signal dilution corresponds to the number of replications for up to 10 generations. This approach revealed that many bacteria internalized by macrophage cells do not replicate, but appear to enter a dormant-like state which could represent an important reservoir of persistent bacteria in the macrophage model of infection (Helaine et al., 2010).

Another single-cell method of tracking intracellular bacterial replication was developed by McQuate et al. (2017) using long-term (17 h) live-cell imaging of infected cells and subsequent image analysis methods to quantify fluorescent signal expressed by internalized bacteria. This image analysis pipeline approach was applied to track bacterial replication within the SCV in epithelial cells as well as to quantify vacuolar replication vs. survival in macrophages. Consistent with Helaine et al. this long-term imaging method revealed a persistent non-replicating population of *Salmonella* in macrophages. Additionally, the growth of replicating bacterial populations in both epithelial cells and macrophage cells were shown to be diverse and fell into three major categories of: (1) delayed initiation of growth, (2) steady growth that plateaued over time, or (3) consistent, steady growth. The role of the individual effector proteins SteA and SseG in impacting these growth parameters was shown to differ between epithelial cells and macrophages, suggesting that effector proteins may play different roles in infection that depend on the type of host cell and/or the infection model (acute vs. systemic infection; McQuate et al., 2017).

Single cell studies in cultured epithelial cells have recently revealed that *Salmonella* has a bimodal lifestyle consisting of subpopulations of vacuolar and cytosolic bacteria (Knodler, 2015). Escape from the *Salmonella*-containing vacuole results in transcriptional reprogramming of bacteria leading to robust replication in the cytosol. Due to the high number of hyper-replicating bacteria in these cells, however, it is difficult to enumerate the subpopulations using microscopy. To determine the proportion of vacuolar verses cytosolic populations, Knodler et al. (2014) applied a chloroquine resistance assay that relies on differential intracellular distribution of antibiotics in mammalian cells. The weak base chloroquine selectively accumulates to high concentrations within endosomes damaging vacuolar bacteria without accessing cytosolic bacteria and enabling quantification of exclusively cytosolic bacteria by CFU. This study revealed that T3SS-1 is necessary for vacuole escape and that cytosolic bacteria represent more than half of the entire intracellular population.

## Summary and future directions

This review highlights techniques that have been developed to explore the role of individual effector proteins in shaping the complex and dynamic landscape between *Salmonella* and host cells. By recognizing the strengths and limitations of each technique, researchers can apply complementary approaches to uncover the role of effector proteins in influencing bacterial fate, the timing and kinetics of effector protein translocation, and the location and potential target of effector proteins within host cells. While the methods described here have proven to be powerful tools in illuminating the host-pathogen interface, there is room for improvement. A major challenge will be improving the sensitivity of these systems to enable visualization of lowly expressed effector proteins as well as expanding methods to monitor multiple effectors at once. Improved dynamic tracking of effector proteins throughout the infection process will enable us to resolve when, where and how effectors interface with host factors in a single experiment. As technology improves, the push toward high-content approaches to study infection will begin to unravel the complex functional hierarchies orchestrated by effectors during interaction with host cells.

## Author contributions

AY and AP outlined the manuscript; AY wrote the manuscript; AY and AP edited the manuscript.

### Conflict of interest statement

The authors declare that the research was conducted in the absence of any commercial or financial relationships that could be construed as a potential conflict of interest.
